# Joint Assessment of Equilibrium and Neuromotor Function: A Validation Study in Patients with Fibromyalgia

**DOI:** 10.3390/diagnostics10121057

**Published:** 2020-12-06

**Authors:** Rafael Lomas-Vega, Daniel Rodríguez-Almagro, Ana Belén Peinado-Rubia, Noelia Zagalaz-Anula, Francisco Molina, Esteban Obrero-Gaitán, Alfonso Javier Ibáñez-Vera, María Catalina Osuna-Pérez

**Affiliations:** 1 Department of Health Sciences, University of Jaén, 23071 Jaén, Spain; rlomas@ujaen.es (R.L.-V.); nzagalaz@ujaen.es (N.Z.-A.); fjmolina@ujaen.es (F.M.); eobrero@ujaen.es (E.O.-G.); ajibanez@ujaen.es (A.J.I.-V.); mcosuna@ujaen.es (M.C.O.-P.); 2 AFIXA Fibromyalgia Association, 23008 Jaén, Spain; abpr0003@red.ujaen.es

**Keywords:** balance control, dizziness, fibromyalgia, chronic fatigue syndrome, reproducibility of results, disability evaluation

## Abstract

Objective: To develop and validate a tool for evaluating balance and neuromotor function in patients with fibromyalgia (FMS). Methods: Brainstorming, the nominal group technique, and pilot-testing were used to select a battery of 20 functional balance tests that were included in a screening tool. A total of 108 subjects (62 with fibromyalgia syndrome, 22 aged over 65 years, and 24 healthy subjects) participated in this validation study. Factor validity, internal consistency, the ability to discriminate between patients and healthy subjects, and concurrent validity with the Fibromyalgia Impact Questionnaire (FIQ), the Central Sensitization Inventory (CSI), the 12-Item Short-Form Health Survey (SF-12), and other tools for measuring balance, such as the Dizziness Handicap Inventory (DHI), the Activities-Specific Balance Confidence Scale (ABC-16), the Falls Efficacy Scale-International (FES-I), and posturographic parameters, were evaluated. Results: The factorial analysis extracted four factors that explained 70% of the variance. The Alpha Cronbach value was 0.928. Concurrent validity of the screening tool with respect to other tools was high, and the receiver operating characteristic (ROC) curve analysis showed an AUC value of 0.932 for discriminating between healthy and FMS subjects. Severe balance disorder related to head movements in FMS patients was found. Conclusion: The 20-item JAEN (Joint Assessment of Equilibrium and Neuro-motor Function) screening tool is a valid and reliable tool for assessing balance in patients with FMS.

## 1. Introduction

Fibromyalgia syndrome (FMS) is a chronic disease with unknown etiology that is diagnosed based on clinical symptoms [1]. It is characterized by generalized chronic pain that often coexists with fatigue, headache, cognitive deficits, mood disorders, dizziness, joint stiffness, and insomnia [2]. FMS is the second most common rheumatologic disease after osteoarthritis, affecting around 2–8% of the population [3]. Regarding its etiology, it is currently believed that FMS occurs due to abnormal processing of pain and other sensory inputs in the brain, spinal cord and periphery and that it is related to central and peripheral sensitization processes [4].

Several studies have found the presence of a nonspecific balance disorder in patients with FMS generates an increased risk of falls [5,6,7,8,9] and worse results in balance tests than healthy controls [2,5,6,9,10,11,12,13]. The disturbance of balance may depend on the general severity of the condition, because scores on balance tests seem to correlate with measures of quality of life, pain, severity of symptoms [2,6,11], and activities of daily living [13].

The complexity of the systems involved in postural control has made it difficult to detect the origin of the disorder in patients with FMS. Some studies have found worse outcomes for all functional tests as well as for dynamic posturography for patients versus controls [5,6]. Other studies have found worse integration of visual and vestibular information [10], for which other authors have considered possible somatosensory dependence [13]. However, patients with FMS show a higher oscillation speed of the center of pressure (CoP) [11], which has also been related to proprioceptive deficit due to polyneuropathy [14]. In accordance with these findings, several studies have analyzed the effect of balance exercises for patients with FMS [9,15,16] and have achieved contradictory results, possibly due to the heterogeneity of the applied programs for the functional status of the patients, as well as the tools used for outcome measurements.

Postural balance is a complex physiological phenomenon that requires participation of the visual, vestibular, and somatosensory systems. Within each of these systems, there are several organs that can fail and act as the origin of the loss of balance. Classic tests generally challenge the functioning of one of these organs—for example, the Head Shaking Test (HST) analyzes the asymmetry of vestibular function [17], and the Head Impulse Test (HIT) tests the integrity of the Vestibulo-Ocular Reflex [18]. However, when these tests are used in isolation, only a partial view of a very complex aspect, such as postural control, is obtained. Secondly, functional scales designed to conduct a more complete measurement of balance are focused on measuring output without being able to use it to test the specific functioning of each physiological phenomenon [19]. On the other hand, instrumented evaluation, such as computerized dynamic posturography, can isolate global functioning at the vestibular, ocular, or somatosensory level, but it is not a technology that is available to all healthcare centers [20].

As noted, FMS is a health problem for which the cause is unknown and is manifested by a long series of signs and symptoms, of which one of the most disabling is balance deficit. As the cause of FMS is unknown, the pathophysiology that leads to loss of balance in these patients is unknown. Therefore, the development of a screening tool for the different systems that participate in postural control is desirable. The objective of this study is twofold. First, to develop and validate a screening tool for the functioning of the systems involved in postural control, and second, to apply this screening tool in patients with fibromyalgia. This may provide an opportunity to typify the balance disorder suffered by these patients, which may have important implications for clarifying the pathophysiology of the disease itself.

## 2. Materials and Methods

### 2.1. Study

To meet the objectives stated above, first, the study used the brainstorming and nominal group technique to select items, develop responses, and determine the level of disability for each response alternative. A total of 10 experts with more than 10 years of experience in the field participated in this preliminary phase. A pilot study followed by a cross-sectional observational study was carried out to validate the screening tool.

### 2.2. Tool Development and Pilot Study

In the first phase of the study, the team aimed to create a battery of screening tests for the different aspects of balance in people with different degrees of disability due to physical or neuro-motor deterioration. To design and develop the items included in this new screening tool, a detailed review of the literature was carried out, and a total of 10 experts with more than 10 years of experience in the field were invited to participate in this process during three sessions of one hour. These sessions dealt with static balance, vestibular tests, and dynamic balance and gait. Brainstorming techniques were used to develop a valid battery of tests, and the nominal group technique was used to obtain a statement of the items. The nominal group technique consisted of a phase where the moderator presented the problem, a phase of individual reflection in which each proposed item was evaluated by the experts, scoring the degree of relevance from 1 to 4 (1 being “not relevant” and 4 being “very relevant”), and a final discussion and consensus phase. Agreement was reached for the order of the items in a logical presentation, the level of disability for each item, and the different response alternatives. We decided to develop responses following the generic qualifier of an International Classification of Functioning (ICF) code, which requires qualifiers to denote the magnitude or severity of the problem in question. According to this, the answer options and their respective scores were no balance problems (0 points), mild problems (1 point), moderate problems (2 points), severe problems (3 points), and complete problems (4 points). A pilot study in ten patients with FMS was carried out to test the selection of items, the five levels of answer classification, and to check whether adjustments were necessary. 

### 2.3. Sample of Patients

The minimum sample size requirement was set at 100 patients; this is the minimum sample size needed to perform a factorial analysis, according to Kline’s criteria [21]. Three types of population with different degrees of balance disorder were needed to validate and test the screening tool: patients diagnosed with FMS, elderly people, and healthy volunteers. Patients with FMS were recruited from the Fibromyalgia Association of Jaen city (AFIXA), and they had to meet the following inclusion criteria: (1) aged > 18 years and (2) fully met the diagnostic criteria for fibromyalgia, as described by the 2016 American College of Rheumatology (ACR). Older adults, recruited from a university for older students, needed to meet the following inclusion criteria: (1) healthy, (2) over 65 years old, and (3) have the capacity to communicate and understand the instructions. Healthy controls were recruited via adverts on different social networks and needed to meet the following inclusion criteria: (1) aged > 18 years, (2) healthy, and (3) female (to balance the sample with FMS patients in terms of age and sex). The final sample was composed of 108 subjects, of whom 62 were patients with FMS, 24 were healthy controls with a similar age and sex distribution to the FMS patients, and 22 were healthy subjects over 65 years of age.

This study was designed following the principles outlined in the Declaration of Helsinki and was approved by the Ethics Committee of Jaen University (ethic approval code: ABR.19/6.TFM). Participants were provided with detailed information regarding the project. Next, written informed consent was obtained from each participant, indicating their voluntary acceptance of participation in the study.

### 2.4. Measurements

Sociodemographic characteristics and anthropometric variables were collected, including sex, age, body mass index (BMI), and education level.

The number of falls in the last 12 months was recorded by asking participants to answer the following question: “How many falls have you suffered in the last year?”

The impact of the disease in the FMS population was measured with the Spanish version of the Fibromyalgia Impact Questionnaire (FIQ) [22]. The FIQ is composed of 10 items that measure pain, rigidity, fatigue, depression and anxiety, disability, and general well-being during the last week. Each symptom is measured on a response scale of 0 (absence of symptoms) to 10 (very severe). The FIQ total score ranges from 0 to 100, where higher values indicate a greater negative impact of the disease.

The presence of central sensitization was measured in the FMS population with the Spanish version of the central sensitization inventory (CSI) [23]. The CSI includes 25 items and evaluates a wide range of somatic and emotional symptoms common to central sensitization syndromes (CSS). The total score ranges from 0 to 100, where higher scores indicate greater severity of symptoms. The Spanish version of the CSI demonstrated high internal consistency (α = 0.872) and a one-dimensional factor structure. 

Balance confidence was recorded in the whole cohort using the Spanish version of the Activities-specific balance confidence scale (ABC-16) [24]. This is a 16-item questionnaire. In the ABC scale, each item can be scored from 0% (zero confidence) to 100% (complete confidence). The total score is between 0% and 100%, with higher values associated with greater balance confidence. The Spanish version of the ABC showed excellent internal consistency (Cronbach’s α = 0.916) and a three-dimension factor structure (explained variance was 62.24%).

Fear of falling during daily activities was assessed using the Spanish version of the Falls Efficacy Scale-International (FES-I) in the whole cohort [25]. The FES-I is a short (16 items) questionnaire that measures the level of concern about falling during social and physical activities inside and outside the home, whether or not the person actually does the activity. A higher score on the FES-I is associated with a greater fear of falling. The FES-I has a good internal consistency (Cronbach α = 0.940) and one unifactorial structure with two underlying dimensions related to less or more demanding physical activities.

Disability due to vertigo was measured with the Spanish version of the dizziness handicap inventory (DHI) [26]. This instrument contains 25 self-administered questions, with a total score of 0 to 100 points being possible. A higher score indicates a greater degree of disability due to vertiginous symptoms. Three subscales are identified: emotional, functional, and physical. The DHI is a very useful multi-dimensional tool for quantifying self-perceived disability in patients with vertigo, dizziness, or instability and the impacts of these conditions on activities of daily living. The Spanish version of the DHI has a high level of internal consistency (α = 0.87) [27]. 

The Spanish version of the 12-Item Short-Form Health Survey (SF-12) was used to measure health-related quality of life in the whole cohort [28]. This is a self-administered questionnaire extracted from the SF-36 using multiple regression. The SF-12 consists of 12 items from which the physical and mental component summaries (PCS-12 and MCS-12, respectively) each yield a single score. The SF-12 items explained 91% of the variance of SF-36 summary components [28].

Posturographic parameters were recorded with a posturographic platform with pressure-resistive sensors (Sensor Medica, Rome, Italy) with a 400 × 400 mm surface and an acquisition frequency of 40 Hz using FreeStep© Standard 3.0 software (Sensor Medica, Rome, Italy). This platform has shown a moderate to substantial reliability for measuring posturographic parameters [29]. In this process, the patient stands on the platform. Their feet should be bare and form a 30° angle, with 2 cm of separation between the heels, and arms relaxed, extended, and touching both sides of the body. Eyes should be looking horizontally at a fixed point on the wall, situated 2.5 m from the platform. The duration of each test was 60 s, during which the patient was asked to remain relaxed and immobile. There was a 60 s interval between each test, and tests involved having both eyes open and eyes closed. For each test, the posturographic parameters recorded related to the participants’ CoP under each condition were as follows: the area covered by the CoP (S, mm^2^); the velocity of CoP movement (V, mm/s); two CoP dispersion parameters, namely the root mean square amplitude of CoP in the mediolateral (RMSX) and anteroposterior (RMSY) directions (mm); and two CoP position parameters, namely the mean CoP on the X axis and the mean CoP on the Y axis.

### 2.5. Data Analysis

Data handling and analysis were conducted using the statistical package for social sciences (SPSS) version 21 (SPSS Inc., Chicago, IL, USA). A level of confidence of 95% was used (*p* < 0.05). Construct validity was assessed using an exploratory factorial analysis of the scores of the screening tool items through a principal component analysis with Varimax rotation. To test the suitability of the sample to perform the factorial analysis, the Kaiser–Meyer–Olkin (KMO) test was employed. 

Cronbach’s α coefficient was used to assess the internal consistency of the instrument. Values between 0.70 and 0.95 were considered acceptable [30]. Concurrent validity was obtained by comparing the screening tool with the Spanish versions of the ABC, FES-I, SF-12, and DHI scales, as well as with the number of falls during the last 12 months and measures of central sensitization and the impact of fibromyalgia. The Pearson or Spearman correlation coefficient was used to analyze the total score obtained by the screening tool with the rest of the measures. A correlation coefficient between 0.3 and 0.5 indicated moderate correlation, while values greater than 0.5 indicated a strong correlation [31].

The accuracy of the screening tool total score in discriminating patients with and without FMS (in terms of balance disorder in fibromyalgia syndrome), between healthy controls and older adult subjects (in terms of age-related balance disturbances), and between subjects who had experienced or not experienced falls during the last 12 months was evaluated using receiver operating characteristic (ROC) curve analysis. In a ROC curve, the true-positive rate (sensitivity) is plotted as a function of the false-positive rate (100-specificity) for different cut-off points. Each point on the ROC curve represents a sensitivity/specificity pair corresponding to a particular decision threshold [32]. A test with perfect discrimination would have a ROC curve passing through the top left-hand corner (100% sensitivity and 100% specificity). We calculated the area under the ROC curve (AUC) as a measure of how well the screening tool total score could distinguish between healthy and FMS patients, healthy and older adult subjects, and subjects who had or had not experienced falls in the last 12 months. The AUC value was considered statistically significant when the 95% CI did not include a value of 0.5. The method developed by Hanley and McNeil [33] was used to calculate the standard error of the AUC, and the binomial exact test was used to calculate the CI for the AUC.

Finally, to test the functional differences regarding balance between FMS patients, healthy individuals, and older adult subjects, a one-way analysis of variance (ANOVA) was employed, where the mean scores of the three populations (FMS, healthy, and older adult subjects) were compared for each factor of the 20-item screening tool.

## 3. Results

### 3.1. Instrument Development Phase

In the first phase of the study, through the brainstorming and nominal group sessions, a battery of 30 eligible items was obtained. This battery of 30 tests included in the Joint Assessment of Equilibrium and Neuro-motor Function (which has been called the JAEN screening tool) is shown in Supplementary Materials. Secondly, we applied the battery of tests to 10 patients with FMS and carefully registered the reactions. Third, the team analyzed the results and checked that the ten items did not have sufficient variability in patient response; five of them were easier for the majority of patients, and the other five items were too difficult to conduct with success. Then, the team slightly adjusted the response categories based on the responses obtained with the pilot study, and, finally, a 20-item JAEN screening tool version (Table S1) was obtained for the validation phase. This instrument contains 20 functional balance tests with alternative answers classified in five categories ranging from no balance problem (0 points) to a complete or total balance problem (4 points). The total scale ranges are from 0 to 80 points. A higher score indicates a greater degree of balance disorder. The performance of all the tests only requires a stopwatch, and the average time to implement the test battery is 12–13 min.

### 3.2. Validation Phase

The sociodemographic and basic data for the sample are shown in Table 1.

The factorial analysis by principal components showed a good KMO measure (KMO = 0.879, Chi^2^ = 1627.348, *p* < 0.001), which means the sample of patients could be considered adequate for the analysis. This analysis extracted four factors that explained 70% of the variance (Table 2).

Table 3 shows that the varimax rotation grouped the items into four following recognizable factors: instability during head movements (balance related to head and neck movement); instability when support is reduced; instability during gait with eyes open and instability during standing and walking with eyes closed. The item with the poorest contribution was the Babinski–Weil Walk item.

The analysis of internal consistency showed a good Alpha Cronbach value of 0.928. Table 4 shows the analysis of the items. Again, the poorest correlation was obtained for the Babinski–Weil Walk. The elimination of each item did not improve the alpha value.

The concurrent validity of the 20-item JAEN screening tool with respect to other tools for measuring balance, such as the DHI, ABC, and FES, was high (Table 5), and it was moderate with respect to the number of falls in the last year. The 20-item JAEN screening tool significantly correlated with the CSI and Physical Component Summary with good concurrent validity. The concurrent validity of the 20-item JAEN screening tool with respect to other impact measures, such as the FIQ or Mental Component Summary SF-12, was moderate, and the correlation was high with respect to some posturography parameters (Table 6) such as the area covered by the CoP (both with eyes open and closed), was moderate respect the root mean square amplitude of CoP in the mediolateral (RMSX) direction (eyes closed) and was small respect the velocity of CoP movement (eyes closed) and the root mean square amplitude of CoP in the anteroposterior (RMSY) direction (eyes closed).

**Figure 1:**
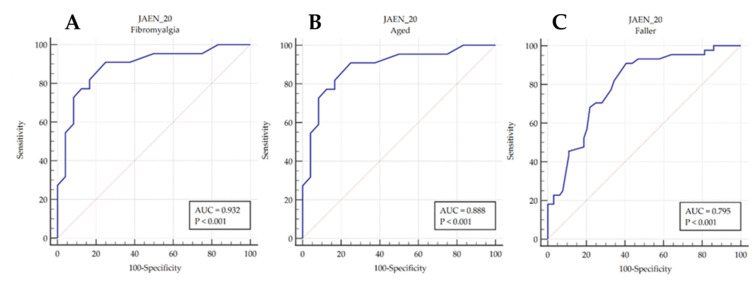
Receiver operating characteristic (ROC) curve to discriminate between fibromyalgia syndrome (FMS) patients and healthy controls (**A**), older adults and healthy controls (**B**), and fallers and non-fallers (**C**), in terms of balance disorders.

The ROC curve analysis showed an AUC value of 0.932 (CI = 0.857 to 0.975, *p* < 0.001) when discriminating between FMS patients (in terms of balance disorders) and healthy controls, an AUC value of 0.888 (CI = 0.760 to 0.962, *p* < 0.001) when discriminating between older adults and healthy controls, and an AUC of 0.815 when discriminating between fallers and non-fallers (Figure 1).

The ROC curve analysis showed that cut-off values of more than 21, 22, and 25 points in the 20-item JAEN screening tool were able to discriminate between healthy and older adults, fibromyalgia subjects, and fallers and non-fallers subjects, respectively. Predictive values and cut-off points are shown in Table 7.

**Figure 2:**
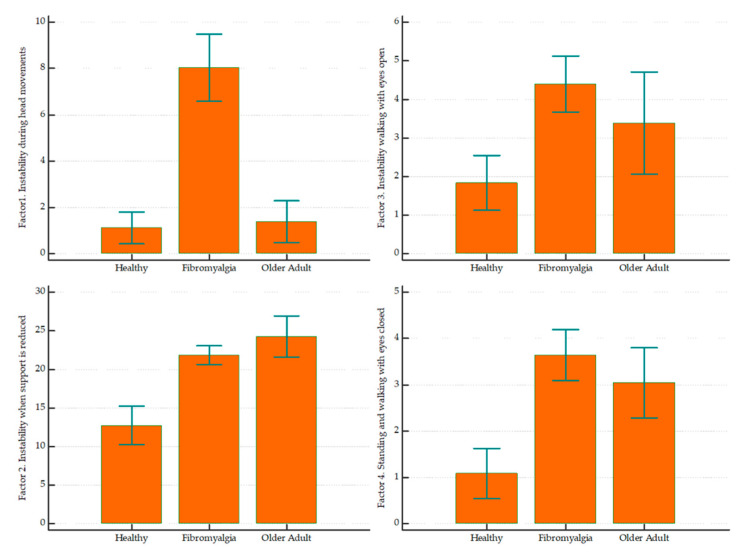
Mean differences between FMS patients, healthy individuals, and older adult subjects for each factor of the 20-item JAEN screening tool. Error bars indicate 95% confidence interval for the mean.

Finally, the ANOVA showed statistically significant differences between groups for each factor in the 20-item JAEN screening tool (Table 8). The analysis revealed that FMS patients and older adult subjects had worse static balance through an unstable stance (Factor 2). In addition, the results show that gait during unsteadiness conditions with open eyes or closed was worse in FMS patients and older adults than in healthy subjects (Factor 3 and 4). In Figure 2, it is possible to appreciate that subjects with FMS present greater balance disturbance related to head and neck movements in the transverse and sagittal planes than healthy and older adult subjects (Factor 1).

## 4. Discussion

This work proposed the development and validation of an instrument composed of a battery of functional tests that can serve as a screening tool for the classification and quantification of balance disorders in patients with FMS. There are currently several tools composed of functional tests that are used to measure balance in different patient populations [34,35]. These tools have several advantages as they have shown good ability to predict the risk of falls in older adults [36]. However, the contribution of these instruments to the screening and classification of balance disorders has not been sufficiently studied. To our knowledge, the Berg Balance Scale (BBS) has been used a few times in patients with FMS [37,38,39], and the BESTest has not yet been used. Therefore, the contribution of these test batteries to the understanding of the pathophysiology that underlies the loss of balance in patients with FMS has been null.

The reason why the BESTest has not been used in patients with FMS may be the fact that it involves performing 36 tasks with an average duration of 30–35 min [40]. This can be difficult for patients suffering from fatigue, such as FMS patients, to complete. The improvement that our tool introduces is that it involves the completion of 20 tasks that are completed in 12–15 min; hence, we think it is more convenient for patients with SF.

The screening tool developed in this work can quantify balance disorder, such as BBS, without any material requirement, and it can analyze systems that are altered in less time and with less effort than the BESTest Score and at a much lower cost than the Dynamic Posturography Computerized (PDC) assessment. In fact, in terms of the greater involvement of the visual and vestibular spheres in patients with FMS, our results are similar to those obtained with the latest technology. However, while the PDC requires the use of a highly equipped laboratory, our tool can be implemented in any cabinet of an association or in primary care centers.

After a pilot study with ten patients, a final 20-item version of the JAEN screening tool was obtained. The analysis of the final version of the 20-item JAEN screening tool showed a factorial structure composed of four factors, good internal consistency, high correlations with posturography parameters, and moderate to strong correlations for the concurrent validity analysis of the questionnaire. Therefore, the ROC curve analysis indicated that the present tool has the capacity to discriminate between healthy individuals and fibromyalgia patients and older adult subjects and between fallers and non-fallers. The subjects of this study had characteristics similar to those of other studies that validated balance measurement tools [35,40], and the group of patients with FMS conformed to the standard profile in terms of sociodemographic characteristics [41].

The results of the principal component analysis show a clear factorial structure composed of four factors that explained more than 70% of the variance. The first factor was composed of items that imply cephalic movements in both the transverse and sagittal plane, with opened and closed eyes, and the integrity of the cervico-ocular and vestibulo-ocular reflexes were evaluated as well as the functionality of the semicircular canals. The second-factor cluster of items assessed static balance through an unstable stance with both opened and closed eyes. It jointly evaluated the propioceptive and visual contributions to body stability. The third factor brought together the items that assess gait in unsteady conditions with open eyes, allowing the evaluation of dynamic balance with opened eyes. Lastly, the fourth factor involved items that evaluate gait with closed eyes, allowing the evaluation of dynamic balance with closed eyes.

The reliability analysis for the 20-item JAEN screening tool showed good internal consistency (α = 0.928), in line with the alpha’s Cronbach results reported by classic screening tests [24,25,26]. In addition, the analysis of concurrent validity showed strong correlation values, not only with all functional, impact, and balance scales, but also with some important posturographic parameters. With the latter, the 20-item JAEN screening tool was correlated with the sway area with both opened and closed eyes, the sway velocity with closed eyes, RMSX with closed eyes, and RMSY with closed eyes. All of these posturographic parameters have been previously established under closed-eyed conditions as significant predictors of fall risk [42], a fact that supports the good results obtained by the present tool to predict the risk of falling (Table 7). In this respect, the 20-item JAEN screening tool has also demonstrated high levels of sensitivity (90.32%) and specificity (83.33%) for identifying FMS patients, with high positive and negative predictive values (+PV = 93.3; −PV = 76.9). This may be explained by the low balance confidence level and the elevated risk of falling reported by FMS patients [43], which are likely associated with the impairments that this population suffers at the vestibular and somatosensory levels, as well as postural reflex disturbances [7]. Moreover, this tool has a great capacity to identify patients who have experienced falls, showing a sensitivity of 90.91% and a specificity of 59.38%. 

The results of the analysis of the clinimetric properties of the 20-item JAEN screening tool show good results that certify this function-centered tool as a valid and reliable method to evaluate balance. In addition, this tool has demonstrated a great capacity to discriminate between healthy and older adults, fibromyalgia subjects, and subjects who have experienced falls, respectively.

Balance impairments are well-known among older adults, with at least one-third of older adults falling at least once a year [44]. Some studies [45,46] have found that subjects with FMS have significant decreases in the volume and density of the Central Nervous System (CNS) gray matter, specifically in regions related to pain processing (cingulate, insular, and prefrontal cortices) and stress (parahippocampal gyrus). This loss has been shown to be greater than that observed in healthy older subjects, so it has been suggested that FMS could be caused by premature aging of the CNS. In our study, it was found that subjects with FMS presented with a balance disorder similar to that of subjects in the comparison group of older adults, except in head movements, during which patients with FMS presented with a very important alteration compared to both control subjects and older adults.

One of the main study results was balance alterations when patients with FMS made cephalic movements in the transverse and sagittal planes. This may be not only due to dysfunction of the semicircular canals but also due to alterations in the integration of eye stabilization reflexes. Many neurophysiological connections exist between the vestibular, visual, and somatosensory systems. The vestibulo-ocular reflex (VOR) receives positional information from vestibular structures, while the cervico-ocular reflex (COR) receives information from the movements of upper cervical structures, and both work together, in conjunction with visual inputs, to coordinate both head–eye movements and postural stability [47]. These two reflexes should always maintain a balance; thereby, when VOR increases, COR decreases, and vice versa. It is possible to observe this in older adults, where there is an increase in COR because of a reduction in VOR [48] due to structural degeneration of the vestibular system [49]. An imbalance between these reflexes has been observed in patients with whiplash-associated disorders—COR is augmented, due to the associated cervical sensorimotor impairment, while VOR remains stable, which may cause balance impairment [50]. A similar process could be occurring in the case of patients with FMS, in whom the VOR could be increased, as a cause of decreased integration of visual and vestibular inputs [10], whereas the COR remains stable.

In line with previous studies where postural control impairments were reported, our results not only show that static balance in unstable conditions and balance with closed eyes is similar in FMS patients and older adults, but also that these individuals have an impairment in dynamic balance in comparison with healthy subjects. To ensure postural control under any stability conditions, the central nervous system implements two different and complementary mechanisms, anticipatory postural adjustment (APA) and compensatory postural adjustment (CPA), where the visual and somatosensory systems play a major role [51]. Anticipatory postural control is mainly based on previous experience, where cognitive capacity affects predictive control and real-time processing of sensory information input [52]. In this regard, important cognitive damage has been found in FMS patients, and it is possible to observe a decreased processing speed and difficulties in execution functions [53], as well as increased displacement of the center of pressure [11], which may lead to the use of different strategies for maintaining balance when standing than those used by healthy subjects, and these are associated with a negative impact on functional independence [13]. Moreover, deficits in the integration of visual and vestibular inputs in this population have been found [10]. In line with the above information, our study results may lead one to think that subjects with FMS could present deficits in the integration of visual, vestibular, and somatosensory inputs, as well as in their cognitive capacity, which could lead to distorted spatial perception. In this way, anticipatory and compensatory postural adjustments could be hampered, generating an increase in the oscillation of the pressure center and, therefore, greater instability.

While the present study presents interesting and relevant outcomes, several limitations are present in it. Although the study sample was large enough to carry out the statistical analysis, the number of subjects was too low to carry out a thorough analysis, which a tool of this relevance deserves. In addition, the clinimetric properties of the 20-item JAEN screening tool were only analyzed in healthy individuals, older adults, and FMS subjects, and it is therefore necessary to explore these properties in a greater number of patients with different pathologies related to balance disorders. Another limitation is the absence of an inter and intra-observer reliability analysis; this should be analyzed in the future. Conversely, it would be interesting to develop future studies in which the clinimetric properties of the 20-item JAEN screening tool are analyzed in patients with different balance disorders, and inter and intra-observer reliability analyses should be explored. Moreover, due to the greater balance affectation found in the present research when FMS patients performed cephalic movements in the transverse and sagittal planes, it would be interesting to observe this effect in patients with FMS undergoing a vestibular rehabilitation program with a VOR desensitization process through gradual exposure to the cephalic movement affected.

## 5. Conclusions

The 20-item JAEN screening tool is a valid and reliable function-based tool for assessing balance in patients with FMS. This instrument contains 20 functional balance tests with responses classified into five categories from no balance problems to a complete or total balance problem. The total score ranges from 0 to 80, with a higher score indicating a greater degree of balance disorder.

The screening tool shows high internal consistency and a factorial structure that explains more than 70% of the variance and comprises the following four well-defined factors: instability during head movements, instability when support is reduced, instability during gait with eyes open, and instability during standing and walking with eyes closed. The 20-item JAEN screening tool shows a strong correlation with other tools used for measuring balance, including the DHI, ABC-16, and FES-I, and also a significant correlation with respect to posturography parameters, such as the sway area, the velocity and the root mean square amplitude of the CoP in both the mediolateral and anteroposterior directions with eyes closed. 

This tool has the capacity to discriminate between healthy individuals and fibromyalgia subjects and between healthy individuals and older adult subjects with high levels of sensitivity and specificity in terms of the presence of balance disorders associated with these populations. It also has the capacity to predict the risk of falling, which is indicated when the screening tool score is greater than 25. 

The results show that static balance and gait during unsteadiness conditions with open or closed eyes is significantly worse in fibromyalgia patients and older adults than in healthy subjects. Severe balance disorder related to head and neck movements was also found in FMS patients, which constitutes an important finding that could open a new line of research in this field. 

## Figures and Tables

**Table 1:** Descriptive characteristics for the whole cohort and for the three populations separately.

Variables	Total Cohort (*n* = 108)	Fibromyalgia (*n* = 62)	Older Adult (*n* = 22)	Healthy (*n* = 24)	*p*-Value
Sex Female Male	101 (93.5) 7 (6.5)	57 (91.9) 5 (8.1)	20 (90.9) 2 (9.1)	24 (100) 0 (0)	0.338
Education level None Primary Secondary University	5 (4.9) 26 (25.2) 35 (34) 37 (35.9)	2 (3.2) 19 (30.6) 26 (41.9) 15 (24.2)	3 (17.6) 1 (5.9) 5 (29.4) 8 (47.1)	0 (0) 6 (25) 4 (16.7) 14 (58.3)	0.003
Age (years)	57.62 ± 9.35	55.74 ± 7.75	69 ± 4.76	52.04 ± 7.80	0.001
BMI (kg/m^2^)	28.07 ± 5	28.53 ± 5.12	29.72 ± 4.89	25.83 ± 4.14	0.029
Falls (last year)	0.91 ± 1.78	1.19 ± 1.25	0.95 ± 3.25	0.13 ± 0.33	0.043
DHI Emotional	10.06 ± 9.76	15.52 ± 8.41	3 ± 5.58	2.42 ± 6.18	0.001
DHI Functional	13.18 ± 10.94	19.24 ± 8.81	6 ± 8.81	4.08 ± 6.71	0.001
DHI Physical	14.31 ± 8.91	19.34 ± 5.51	10.36 ± 8.89	4.92 ± 6.46	0.001
DHI TOTAL (0–100)	37.54 ± 27.76	54.10 ± 20.77	19.36 ± 19.34	11.42 ± 18.26	0.001
ABC (0–100)	69.34 ± 23.35	57.99 ± 22.4	80.38 ± 12.43	89.43 ± 12.49	0.001
FES-I	29.14 ± 10.7	34.27 ± 10.5	23.05 ± 5.44	21.21 ± 6.29	0.001
FIQ (0–100)	Not applicable	68.02 ± 16.17	Not applicable	Not applicable	Not applicable
CSI (0–100)	Not applicable	61.94 ± 10.82	Not applicable	Not applicable	Not applicable
Physical SF-12	38.25 ± 11.49	30.62 ± 5.59	48.43 ± 8.46	49.9 ± 8.62	0.001
Mental SF-12	38.55 ± 12.52	32.68 ± 10.8	45.62 ± 9.85	48.13 ± 9.67	0.001

Data are given as mean ± SD for continuous variables and frequencies (percentages) for categorical variables. One-way ANOVA was used to analyze the distribution of the quantitative variables and the chi-squared test for categorical variables. ABC: activities-specific balance confidence scale; BMI: Body Mass Index; CSI: central sensitization inventory; DHI: dizziness handicap inventory; FES: Falls Efficacy Scale-International; FIQ: Fibromyalgia Impact Questionnaire; SF-12: 12-Item Short-Form Health Survey.

**Table 2:** Explained variance of the 20-item Joint Assessment of Equilibrium and Neuro-motor Function (JAEN) screening tool items by principal component analysis.

Component	Initial Eigenvalues			Sums of the Squared Saturations of the Extraction			Sum of the Squared Saturations of the Rotation		
	Total	% of Variance	% Accumulated	Total	% of Variance	% Accumulated	Total	% of Variance	% Accumulated
1	8.570	42.848	42.848	8.570	42.848	42.848	4.760	23.798	23.798
2	3.340	16.701	59.549	3.340	16.701	59.549	4.537	22.683	46.482
3	1.252	6.262	65.811	1.252	6.262	65.811	2.471	12.353	58.834
4	1.011	5.057	70.868	1.011	5.057	70.868	2.407	12.034	70.868
5	0.928	4.642	75.510						
6	0.775	3.874	79.384						
7	0.583	2.917	82.301						
8	0.546	2.729	85.030						
9	0.501	2.507	87.537						
10	0.403	2.017	89.554						
11	0.354	1.768	91.322						
12	0.343	1.713	93.035						
13	0.298	1.491	94.526						
14	0.239	1.195	95.722						
15	0.215	1.074	96.796						
16	0.189	0.947	97.743						
17	0.146	0.729	98.471						
18	0.130	0.648	99.120						
19	0.114	0.570	99.690						
20	0.062	0.310	10 < 0.001						

**Table 3:** Rotated component matrix of the 20-item JAEN screening tool obtained by principal component analysis with Varimax rotation.

	Component			
	Instability during Head Movements	Instability When Support Is Reduced	Instability during Gait with Eyes Open	Instability Standing and Walking with Eyes Closed
Standing Eyes Closed (Romberg test)				0.695
Standing Tandem Left		0.539		
Tandem Romberg Left		0.583		
Standing Tandem Right		0.628		
Tandem Romberg Right		0.560		
The One-Legged Stance Time Left Eyes Open		0.864		
The One-Legged Stance Time Left Eyes Closed		0.814		
The One-Legged Stance Time Right Eyes Open		0.830		
The One-Legged Stance Time Right Eyes Closed		0.804		
Modified Head Shaking Rotation Test Eyes Closed	0.832			
Modified Head Shaking Flexion Test Eyes Open Left	0.859			
Modified Head Shaking Flexion Test Eyes Closed Left	0.868			
Modified Head Shaking Flexion Test Eyes Open Right	0.868			
Modified Head Shaking Flexion Test Eyes Closed Right	0.881			
Sphinx Pose (during 30 s)	0.703			
Fukuda Stepping Test 50 Steps				0.701
Babinski–Weil Walk				0.349
Walk tandem Eyes Open			0.624	
Walk Shaking Neck Flexion–Extension Eyes Open			0.800	
Walk Shaking Neck Rotation Eyes Open			0.777	

**Table 4:** Item analysis.

	Mean of the Screening Tool If the Item Is Deleted	Variance of the Screening Tool If the Item Is Deleted	Corrected Correlation Item-Total	Squared Multiple Correlation	Cronbach Alpha If Item Is Deleted
Standing Eyes Closed (Romberg test)	31.09	173.075	0.609	0.534	0.925
Standing Tandem Left	30.44	164.715	0.697	0.695	0.923
Tandem Romberg Left	29.31	171.975	0.597	0.585	0.925
Standing Tandem Right	30.47	166.364	0.684	0.706	0.923
Tandem Romberg Right	29.45	169.783	0.594	0.593	0.925
The One-Legged Stance Time Left Eyes Open	29.58	169.909	0.612	0.796	0.925
The One-Legged Stance Time Left Eyes Closed	28.81	175.055	0.533	0.646	0.926
The One-Legged Stance Time Right Eyes Open	29.51	169.766	0.587	0.701	0.925
The One-Legged Stance Time Right Eyes Closed	28.78	174.231	0.582	0.621	0.925
Modified Head shaking Rotation Test Eyes Closed	31.33	169.738	0.657	0.812	0.924
Modified Head Shaking Flexion Test Eyes Open Left	31.29	168.150	0.653	0.844	0.924
Modified Head Shaking Flexion Test Eyes Closed Left	31.06	166.940	0.679	0.843	0.923
Modified Head Shaking Flexion Test Eyes Open Right	31.31	169.167	0.659	0.851	0.924
Modified Head shaking Flexion Test Eyes Closed Right	31.19	167.330	0.700	0.892	0.923
Sphinx Pose (during 30 s)	31.17	175.206	0.474	0.466	0.927
Fukuda stepping Test 50 steps	31.40	173.887	0.502	0.430	0.927
Babinski-Weil Walk	30.81	175.952	0.421	0.307	0.928
Walk Tandem Eyes Open	30.81	166.040	0.609	0.549	0.925
Walk Shaking Neck Flexion–Extension Eyes Open	31.09	171.150	0.639	0.719	0.924
Walk Shaking Neck Rotation Eyes Open	30.69	170.517	0.592	0.661	0.925

**Table 5:** Correlation between the 20-item JAEN screening tool and functional impact and balance scales.

	20-Item JAEN Screening Tool	
Variable	R	*p*-Value
Number of falls in the last year	0.486	<0.001
Dizziness Handicap Inventory Emotional	0.578	<0.001
Dizziness Handicap Inventory Functional	0.650	<0.001
Dizziness Handicap Inventory Physical	0.698	<0.001
Dizziness Handicap Inventory TOTAL Score (DHI)	0.675	<0.001
Activities Balance Confidence Scale (ABC)	−0.669	<0.001
Falls Efficacy Scale-International	0.581	<0.001
Fibromyalgia Impact Questionnaire TOTAL (FIQ)	0.348	0.003
Central Sensitization Inventory TOTAL (CSI)	0.656	<0.001
Physical Component Summary SF-12	−0.649	<0.001
Mental Component Summary SF-12	−0.370	<0.001

**Table 6:** Spearman correlations between the 20-item JAEN screening tool and posturographic parameters.

Posturographic Parameter	20-Item JAEN Screening Tool	
	Rho	*p*-Value
Sway Area Eyes Open	0.570	<0.001 ***
Velocity CoP Eyes Open	−0.014	0.886
RMSX Eyes Open	0.182	0.062
RMSY Eyes Open	−0.056	0.566
Mean CoP X axis Eyes Open	−0.090	0.357
Mean CoP Y axis Eyes Open	−0.084	0.394
Sway Area Eyes Closed	0.604	<0.001 ***
Velocity Eyes Closed	0.257	0.008 **
RMSX Eyes Closed	0.431	<0.001 ***
RMSY Eyes Closed	0.236	0.015 *
Mean CoP X axis Eyes Closed	−0.143	0.145
Mean CoP Y axis Eyes Closed	−0.171	0.080

20-item JAEN screening tool: Joint Assessment of Equilibrium and Neuromotor Function; CoP: center of pressure; RMSX: root mean squared calculated by X axis position values; RMSY: root mean squared calculated by Y axis position values. * *p* < 0.05; ** *p* < 0.01; *** *p* < 0.001.

**Table 7:** Capacity of the 20-item JAEN screening tool to discriminate between patients and controls: cut-off points and their predictive values.

	Fibromyalgia	95% CI	Older Adult	95% CI	Faller	95% CI
Criterion	>22		>21		>25	
Sensitivity	90.32	80.1–96.4	90.91	70.8–98.9	90.91	78.3–97.5
Specificity	83.33	62.6–95.3	75	53.3–90.2	59.38	46.4–71.5
+LR	5.42	2.2–13.3	3.64	1.8–7.4	2.24	1.6–3.1
−LR	0.12	0.05–0.3	0.12	0.03–0.5	0.15	0.06–0.4
+PV	93.3	85.1–97.2	76.9	62.2–87.1	60.6	53.0–67.7
−PV	76.9	60.4–87.9	90	70.2–97.2	90.5	78.5–96.1

**Table 8:** Between-group differences in factor components of the 20-item JAEN screening tool.

	Healthy		Fibromyalgia		Older Adult		ANOVA
	Mean	SD	Mean	SD	Mean	SD	*p*-Value
Factor 1. Instability during head movements.	1.13	1.62	8.03	5.72	1.39	2.08	<0.001
Factor 2. Instability when support is reduced	12.75	5.90	21.84	4.69	24.27	5.93	<0.001
Factor 3. Instability during gait with eyes open	1.83	1.69	4.40	2.86	3.39	3.06	0.001
Factor 4. Instability when standing and walking with eyes closed	1.08	1.28	3.65	2.18	3.05	1.70	<0.001

## Supplementary Materials

The following are available online at https://www.mdpi.com/2075-4418/10/12/1057/s1, 20-item JAEN screening tool.
