# Supplementary material for: Joint Assessment of Equilibrium and Neuromotor Function: A Validation Study in Patients with Fibromyalgia
Source: Diagnostics (Basel). 2020 Dec 6;10(12):1057. doi: 10.3390/diagnostics10121057 (PMC7762125; doi:10.3390/diagnostics10121057)
Supplement: Supplementary file 1 [file diagnostics-10-01057-s001.pdf]

THE 20 JAÉN Scale. Joint Assessment of Equilibrium and Neuromotor Status in Patients with Fibromyalgia.

	DISABILITY SCORE ACCORDING TO THE INTERNATIONAL CLASSIFICATION OF FUNCTIONING, DISABILITY AND HEALTH (ICF)					
	NO PROBLEM =0	MILD PROBLEM=1	MODERATE PROBLEM=2	SEVERE PROBLEM=3	COMPLETE PROBLEM =4	SCORE
**STATIC BALANCE TEST**						
Standing Eyes Open (30 seconds)	Straight body position and stable (30 seconds)	Lateral deviation of the body but stable	Instability	Fall / finish before 30 seconds	Fall / finish before 15 seconds	
**1.-Standing Eyes Closed (Romberg test)**	**Straight body position and stable (30 seconds)**	**Lateral deviation of the body but stable**	**Instability**	**Fall / finish before 30 seconds**	**Fall / finish before 15 seconds**	
**2.-Standing Tandem Left**	**Straight body position and stable (30 seconds)**	**Lateral deviation of the body but stable**	**Instability**	**Fall / finish before 30 seconds**	**Fall / finish before 15 seconds**	
**3.-Tandem Romberg Left**	**Straight body position and stable (30 seconds)**	**Lateral deviation of the body but stable**	**Instability**	**Fall / finish before 30 seconds**	**Fall / finish before 15 seconds**	
**4.-Standing Tandem Right**	**Straight body position and stable (30 seconds)**	**Lateral deviation of the body but stable**	**Instability**	**Fall / finish before 30 seconds**	**Fall / finish before 15 seconds**	
**5.-Tandem Romberg Right**	**Straight body position and stable (30 seconds)**	**Lateral deviation of the body but stable**	**Instability**	**Fall / finish before 30 seconds**	**Fall / finish before 15 seconds**	
**6.-One-Legged Stance Time Left Eyes Open**	**Straight body position and stable (30 seconds)**	**Body stabilizing movements but only of the trunk**	**Body stabilizing movements including the load foot**	**Bipodal support before 30 seconds**	**Bipodal support before 15 seconds**	
**7.-One-Legged Stance Time Left Eyes Closed**	**Straight body position and stable (15 seconds)**	**Body stabilizing movements but only of the trunk**	**Body stabilizing movements including the load foot**	**Bipodal support before 15 seconds**	**Impossibility**	
**8.-One-Legged Stance Time Right Eyes Open**	**Straight body position and stable (30 seconds)**	**Body stabilizing movements but only of the trunk**	**Body stabilizing movements including the load foot**	**Bipodal support before 30 seconds**	**Bipodal support before 15 seconds**	
**9.-One-Legged Stance Time Right Eyes Closed**	**Straight body position and stable (15 seconds)**	**Body stabilizing movements but only of the trunk**	**Body stabilizing movements including the load foot**	**Bipodal support before 15 seconds**	**Impossibility**	

VESTIBULAR AND OCULAR RELATED TEST (30 movements with a frequency of 1hz)						
Modified Head Shaking Rotation Test Eyes Open	Straight body position and stable. Without symptoms	Dizziness/ instability at the end. Mild symptoms	Constant Dizziness/ instability. Moderate/severe symptoms	Fall or need to interrupt <30 seconds	Fall or need to interrupt <15 seconds	
**10.-Modified Head Shaking Rotation Test Eyes Closed**	**Straight body position and stable. Without symptoms**	**Dizziness/ instability at the end. Mild symptoms**	**Constant Dizziness/ instability. Moderate/severe symptoms**	**Fall or need to interrupt <30 seconds**	**Fall or need to interrupt <15 seconds**	
**11.-Modified Head Shaking Flexion Test Eyes Open Left (with 45º left rotation)**	**Straight body position and stable. Without symptoms**	**Dizziness/ instability at the end. Mild symptoms**	**Constant Dizziness/ instability. Moderate/severe symptoms**	**Fall or need to interrupt <30 seconds**	**Fall or need to interrupt <15 seconds**	
**12.-Modified Head Shaking Flexion Test Eyes Closed Left (with 45º left rotation)**	**Straight body position and stable. Without symptoms**	**Dizziness/ instability at the end. Mild symptoms**	**Constant Dizziness/ instability. Moderate/severe symptoms**	**Fall or need to interrupt <30 seconds**	**Fall or need to interrupt <15 seconds**	
**13.-Modified Head Shaking Flexion Test Eyes Open Right (with 45º right rotation).**	**Straight body position and stable. Without symptoms**	**Dizziness/ instability at the end. Mild symptoms**	**Constant Dizziness/ instability. Moderate/severe symptoms**	**Fall or need to interrupt <30 seconds**	**Fall or need to interrupt <15 seconds**	
**14.-Modified Head Shaking Flexion Test Eyes Closed Right (with 45º right rotation).**	**Straight body position and stable. Without symptoms**	**Dizziness/ instability at the end. Mild symptoms**	**Constant Dizziness/ instability. Moderate/severe symptoms**	**Fall or need to interrupt <30 seconds**	**Fall or need to interrupt <15 seconds**	
**15.-Sphinx Pose during 30 seconds.**	**Without symptoms**	**Neck discomfort**	**Vegetative symptoms**	**Need to interrupt <30 seconds**	**Need to interrupt <15 seconds**	
Left Peripheral Vision (30 oscillations by the evaluator)	Straight body position and stable. Without symptoms	Dizziness/ instability at the end. Mild symptoms	Constant Dizziness/ instability. Moderate/severe symptoms	Fall or need to interrupt <30 seconds	Fall or need to interrupt <15 seconds	
Right Peripheral Vision (30 oscillations by the evaluator)	Straight body position and stable. Without symptoms	Dizziness/ instability at the end. Mild symptoms	Constant Dizziness/ instability. Moderate/severe symptoms	Fall or need to interrupt <30 seconds	Fall or need to interrupt <15 seconds	
**GAIT**						
Stepping Test 50 Step Eyes Open	Straight position and stable	Instability	Get to finish but with ataxia	Fall or need to interrupt <30 seconds	Fall or need to interrupt <15 seconds	
**16.-Fukuda Stepping Test 50 Step Eyes Closed.**	**Deviation <30º to either side**	**Deviation to one side >30º**	**Get to finish but with ataxia**	**Fall or need to interrupt <30 seconds**	**Fall or need to interrupt <15 seconds**	

Fukuda with Left Cervical Rotation	Right deviation	No deviation	Left deviation	Fall or ataxia before 30 steps	Fall or need to interrupt <15 seconds	
Fukuda with Right Cervical Rotation	Left deviation	No deviation	Right deviation	Fall or ataxia before 30 steps	Fall or need to interrupt <15 seconds	
**17.-Babinski-Weill test (30 steps)**	**Straight position and stable**	**Deviation to one side <30º**	**Deviation to one side >30º**	**Fall or need to interrupt <30 seconds**	**Fall or need to interrupt <15 seconds**	
**18.-Walk tandem Eyes Open (30 seconds)**	**Straight position and stable**	**Get to finish but with instability/ dizziness**	**Get to finish but with ataxia**	**Fall or need to interrupt <30 seconds**	**Fall or need to interrupt <15 seconds**	
Walk tandem Eyes Closed (30 seconds)	Straight position and stable	Get to finish but with instability/ dizziness	Get to finish but with ataxia	Fall or need to interrupt <30 seconds	Fall or need to interrupt <15 seconds	
**19.-Walk Shaking Neck Flexion-Extension Eyes Open (30 seconds)**	**Straight position and stable**	**Get to finish but with instability/ dizziness. Decrease step frequency**	**Get to finish but with ataxia**	**Fall or need to interrupt <30 seconds**	**Fall or need to interrupt <15 seconds**	
Walk Shaking Neck Flexion-Extension Eyes Closed (30 seconds)	Straight position and stable	Get to finish but with instability/ dizziness	Get to finish but with ataxia	Fall or need to interrupt <30 seconds	Fall or need to interrupt <15 seconds	
**20.-Walk Shaking Neck Rotation Eyes Open (30 seconds)**	**Straight position and stable**	**Get to finish but with instability/ dizziness. Decrease step frequency**	**Get to finish but with ataxia**	**Fall or need to interrupt <30 seconds**	**Fall or need to interrupt <15 seconds**	
Walk Shaking Neck Rotation Eyes Closed (30 seconds)	Straight position and stable	Get to finish but with instability/ dizziness	Get to finish but with ataxia	Fall or need to interrupt <30 seconds	Fall or need to interrupt <15 seconds	
**TOTAL SCORE**						
